# Evaluating Active U: an internet-mediated physical activity program

**DOI:** 10.1186/1471-2458-9-331

**Published:** 2009-09-10

**Authors:** Lorraine R Buis, Timothy A Poulton, Robert G Holleman, Ananda Sen, Paul J Resnick, David E Goodrich, LaVaughn Palma-Davis, Caroline R Richardson

**Affiliations:** 1 Wayne State University College of Nursing, 5557 Cass Ave, Detroit, MI 48202, USA; 2 University of Michigan Medical School, 1018 Fuller St, Ann Arbor, MI 48104-1213, USA; 3 Department of Family Medicine, University of Michigan Medical School, 1018 Fuller St, Ann Arbor, MI 48104-1213, USA; 4 Veterans Affairs Medical Center, Center for Clinical Management Research, HSR&D/SMITREC (11H), P.O. Box 130170, Ann Arbor, MI 48113-0170, USA; 5 Center for Statistical Consultation and Research (CSCAR) and Department of Statistics, 3550 Rackham, Ann Arbor, MI 48104-1070 USA; 6 University of Michigan School of Information, 314 West Hall, Ann Arbor, MI 48104-1092, USA; 7 University of Michigan Health & Well-Being Services, MWorks & MFit, 2850 S Industrial #600, Ann Arbor, MI 48104-6773, USA

## Abstract

**Background:**

Engaging in regular physical activity can be challenging, particularly during the winter months. To promote physical activity at the University of Michigan during the winter months, an eight-week Internet-mediated program (Active U) was developed providing participants with an online physical activity log, goal setting, motivational emails, and optional team participation and competition.

**Methods:**

This study is a program evaluation of Active U. Approximately 47,000 faculty, staff, and graduate students were invited to participate in the online Active U intervention in the winter of 2007. Participants were assigned a physical activity goal and were asked to record each physical activity episode into the activity log for eight weeks. Statistics for program reach, effectiveness, adoption, and implementation were calculated using the Re-Aim framework. Multilevel regression analyses were used to assess the decline in rates of data entry and goal attainment during the program, to assess the likelihood of joining a team by demographic characteristics, to test the association between various predictors and the number of weeks an individual met his or her goal, and to analyze server load.

**Results:**

Overall, 7,483 individuals registered with the Active U website (≈16% of eligible), and 79% participated in the program by logging valid data at least once. Staff members, older participants, and those with a BMI < 25 were more likely to meet their weekly physical activity goals, and average rate of meeting goals was higher among participants who joined a competitive team compared to those who participated individually (IRR = 1.28, *P *< .001).

**Conclusion:**

Internet-mediated physical activity interventions that focus on physical activity logging and goal setting while incorporating team competition may help a significant percentage of the target population maintain their physical activity during the winter months.

## Background

Regular physical activity is an important contributor to good health. Increased physical activity has been associated with decreased risk for cardiovascular disease [1-3], type 2 diabetes [4,5], depression [6], and some cancers [7,8]. The American College of Sports Medicine (ACSM) and the American Heart Association (AHA) currently recommend that all Americans engage in physical activity of at least moderate intensity three to five times a week in order to maintain a healthy lifestyle. Engaging in regular physical activity can be challenging, particularly during winter months when individual physical activity levels tend to decline [9-11]. As such, it is important to promote physical activity during the winter when individuals are likely to be less active.

While worksite and community-based interventions increase physical activity and promote weight loss among participants [12-16], they can be expensive to coordinate and often have limited reach. One efficient strategy for reaching large communities of individuals is through the Internet, especially since the majority of Americans now have access to online materials. Recent data from the Pew Internet & American Life project indicates that approximately 71% of adult Americans are Internet users and that approximately 50% have access to broadband Internet at home [17,18]. In addition, some research studies provide evidence that Internet-mediated interventions may be effective for increasing physical activity [19-22].

To help promote physical activity among faculty, staff, and graduate students at the University of Michigan during the winter months, an Internet-mediated program called Active U was developed. Active U is a free intervention that provides participants with an online physical activity log, goal setting, motivational emails, and team competition to encourage physical activity. The purpose of this investigation is to evaluate the Active U program using the Re-Aim framework.

## Methods

### Study Design

Active U is a free, annual, eight-week, Internet-mediated physical activity program developed by the Health Promotion Division of the University of Michigan. This investigation is a program evaluation of Active U using the Re-Aim framework [23,24] and is a retrospective analysis of de-identified data from the 2007 implementation of this intervention. Active U was first implemented in 2006 and has been conducted each subsequent year. Active U is designed to help members of the university community stay active during the winter. In 2007 the program ran from February 6 through April 3 and included the university's spring break, which occurred during the third week of the program. The University of Michigan Institutional Review Board reviewed and exempted the methods utilized in this investigation (UM IRB HUM00012083).

### Participants and Recruitment

In the winter of 2007, approximately 47,074 individuals (6,110 faculty, 26,964 staff, and approximately 14,000 graduate students at the University of Michigan) were invited to participate in the Active U intervention. Participants were recruited from the university community through an intensive advertising program that included mailed program flyers, campus press coverage, website banners, cafeteria table tents, large posters and floor mats with the Active U logo, emails from the Active U team and university administrators, advertisements on city buses, screensavers on core-imaged university computers, a rolling count of enrollment numbers on parking lot light displays, and recruitment from self-selected team leaders. As an incentive for participation, registered participants received an Active U t-shirt, as well as free passes for university fitness facilities for use during the program period. In addition, participants on teams with five or more people were eligible to participate in team competitions. All competitive teams were rewarded with recognition for meeting goals.

### Active U Description

To promote physical activity, Active U utilizes an online, self-reported physical activity-tracking log combined with goal setting, team competition, and weekly motivational emailed newsletters that support continued physical activity. The physical activity log and goal setting components of this program facilitate self-monitoring and self-regulation and are the main theoretically based intervention components [25,26]. Experts in health promotion wrote the newsletter content, which was not limited to a single theoretical framework.

To authenticate eligible University of Michigan faculty, staff, and graduate students, participants registered online for the Active U program by logging on with their university ID and password and filling out a questionnaire assessing baseline levels of physical activity and weight, as well as height, age, employment type, health status, and gender. During the enrollment process, participants had the opportunity to create a new team and to send out email invitations to others to join. Team competitions were introduced to the program to enhance social support and motivation. Those who did not want to start their own team could apply to join an existing team, which required the approval of the team captain. Teams tended to form around pre-existing affiliations such as departments, lab groups, or buildings. In some cases, department and school email lists were used to recruit team members, and it was not unusual for an individual to receive invitations from several different teams, but each participant was only allowed to join one team. Participants were able to track the collective goal attainment of each competitive team of five or more individuals. Competitive teams were ranked according to the average team percentage of goal met for each week. Each week, the teams with the highest percentage of team members meeting their goals were recognized, but no monetary incentives or prizes were given.

At the beginning of the program, participants were assigned an automated physical activity goal expressed as minutes per week of moderate- to vigorous-intensity physical activity. Individuals who self-reported less than 60 minutes of moderate to vigorous physical activity per week at baseline were assigned a physical activity goal of 60 minutes. Individuals who self-reported more than 60 minutes of moderate to vigorous physical activity per week at baseline were assigned a physical activity goal equal to their self-reported baseline amount. Participants had the option to decrease or increase their weekly goal whenever they wanted, as long as the goal was at least 60 minutes per week. During the Active U program, participants recorded each episode of physical activity into the activity log including the type of activity, as well as the minutes of activity. Participants selected activities from a dropdown list with 27 selections and included items such as running/jogging, aerobics, organized sports, cardio equipment, martial arts, dancing, or other moderate- or vigorous-intensity physical activity. Only bouts of activity that lasted for 10 minutes or longer counted toward achieving weekly goals.

Finally, participants received a weekly email containing competitive team rankings, information about the health benefits of physical activity, tips about how to increase and maintain a physically active lifestyle, and a reminder to enter physical activity data into the Active U log.

### Program Evaluation Framework - Re-Aim

The Re-Aim framework is a framework for evaluating interventions that focuses on the potential for an intervention to have an impact outside of a small, controlled research trial. Re-Aim [23,24] has five components: reach, effectiveness, adoption, implementation, and maintenance.

#### Reach

Reach is defined in the Re-Aim theoretical model as the absolute number, proportion, and representativeness of individuals who participate in a given program. Reach is a function of the size of the target population, the number exposed to recruitment, the number who responded to recruitment, the number who are eligible, and the number who participate. Eligible and invited individuals who went to the Active U website and entered their user ID and password and completed a brief survey to register for Active U are counted in the numerator of reach statistics for this intervention. Thus, the reach in this program is the number of individuals who went to the Active U website and registered for the program divided by the number of participants in the target population. We further characterize representativeness of participants based on information from administrative databases and self-reported descriptive variables from the baseline survey.

#### Effectiveness

Effectiveness of Active U is defined as the number of weeks that a participant met his or her physical activity goal. To measure individual weekly goal attainment, the self-reported total minutes of physical activity each week was calculated and compared to an individual's physical activity goal for that week. Possible values for goal attainment ranged from 0 to 8. If individuals failed to enter data for a particular week, it was assumed that they did not meet their weekly physical activity goal. Of particular interest was the degree to which joining a competitive Active U team predicted greater success with meeting weekly goals.

#### Adoption

Adoption in the Re-Aim framework refers to the number of settings and clinicians that successfully adopt the intervention. In an automated Internet-mediated and centrally administered intervention that directly targets participants rather than clinics, hospitals, or providers, adoption can be assessed on an individual level. It is not unusual in Internet-mediated interventions for individuals to sign up for a program but never return after they learn more about the program or to drop out after using it only a few times. To measure adoption, we calculated the number of weeks during the eight-week intervention that each individual participant entered at least one bout of physical activity into the Active U physical activity tracker. The range of possible values for the adoption variable was 0 to 8. In addition to assessing mean adoption across the entire pool of participants, we identified participant characteristics that predicted higher levels of adoption.

Of particular interest was whether or not being a member of a competitive Active U team increased adoption. An analysis of the extent to which participants joined teams and the individual characteristics that predict team joining is presented with results for adoption.

The degree to which an Active U-like program can be disseminated to other universities is another valid measure of adoption, but that is beyond the scope of this paper.

#### Implementation

In the Re-Aim framework, implementation is a construct that primarily addresses variability in the behavior of the intervention provider. For example, implementation may refer to how closely staff members follow the program that the developers provide. However, in an automated computer-delivered intervention the computer delivers the intervention, and barring any bugs in the program, intervention fidelity would be expected to be perfect and every participant would be expected to receive an identical intervention.

Barriers to implementation in Internet-mediated interventions tend to be related to barriers to Internet access. In the university community that is the target population for Active U, daily computer use is a job requirement for most participants and broadband access either at work or home is assumed to be nearly universal. While Internet access was unlikely to be a barrier in this population, Active U server response times may have been a barrier for some. Slow response times in Internet-mediated interventions represent a modifiable problem in implementation, can be discouraging to users, and may impact participation. In Active U, because emailed newsletters were sent out to all participants simultaneously on Tuesday morning reminding them to enter their physical activity data, many users attempted to enter their data at the same time. This caused the intervention website to run slowly making it difficult to enter data. While some users may have tried again later, we hypothesize that some users dropped out because of the slow server response times that they experienced on Tuesday morning. In a server load analysis, server response time was calculated for each day and was measured as the median page load time for the day to estimate difference in load time between days. In addition, we examined dropout rates for users whose last login was on Tuesday vs. those whose last login was any other day of the week, and we hypothesized that dropouts might be higher after a Tuesday login.

#### Maintenance

Active U is a short-term (eight week) intervention, intended to help participants remain active during winter months. An appropriate measure of maintenance in this context may be the extent to which the Active U program continues to be offered every winter as a part of the routine organizational practices and policies of the University. Active U has been offered for the past four years (2006-2009). While the program is well established, the cost of the extensive advertising campaign and participant incentive program remain a threat to maintenance. Cost estimates have been included in the maintenance section of the results. Further analyses of maintenance issues are beyond the scope of this manuscript.

### Measures

#### Demographic and Descriptive Variables

In a baseline online survey, participants self-reported height, weight, and average amount of weekly physical activity in minutes per week. Additionally, participants self-reported categorical demographic data including gender, age (18-29 years, 30-39 years, 40-49 years, 50-59 years, 60-69 years, 70+ years), employment type (faculty, staff, or graduate student), and health status. Health status was measured by a survey item that asked, "In general, how would you describe your health?" Possible responses included *excellent*, *very good*, *good*, *fair*, and *poo*r. A Body Mass Index (BMI) was calculated from self-reported height and weight measures. A few extreme outliers in self-reported height and weight (height < 48 inches and weight > 600 lbs.) that were physiologically unlikely values were treated as missing values. Using the following survey items to determine baseline weekly physical activity, individuals self reporting less than 150 minutes of physical activity per week were classified as sedentary, whereas individuals self-reporting more than 150 minutes of physical activity per week were considered active:

• "In general how many days per week do you engage in *moderate *physical activity such as walking, vacuuming, or gardening?"

• "For every day that you engage in *moderate *physical activity, how many minutes per day do you spend?"

• "In general, how many days per week do you engage in *vigorous *physical activity such as running, bicycling, aerobics, or heavy yard work?"

• "For every day that you engage in *vigorous *physical activity, how many minutes per day do you spend?"

#### Physical Activity Tracker Data

Participants logged into the Active U website and entered their physical activity data throughout the eight-week program. Reported bouts of physical activity < 10 minutes or > 600 minutes were excluded from this analysis and were not counted as valid data entries. It is difficult to accurately track and self-report shorter bouts of physical activity, and some guidelines suggest that bouts of activity lasting less than 10 minutes may not yield the same health benefits as longer bouts of activity [27]. The upper limit of 600 minutes a day represents 10 hours of physical activity in a single day. Values greater than this were extreme outliers and were not included in the analysis.

### Data Analysis

**Figure 1:**
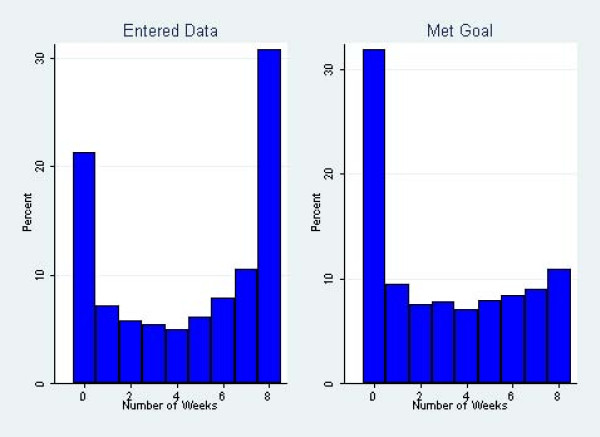
**Histograms of the number of weeks each participant entered data and met goals**.

We conducted data analysis using STATA 10.1. Descriptive statistics including means with standard deviations, frequency tables, and ranges were calculated to describe the reach of the Active U intervention, as well as participation in and adherence to the program. We used multilevel logistic regression analyses adjusting for clustering by team and by individual to assess the decline in rates of data entry and goal attainment during the program. Furthermore, we used logistic regression analysis to assess the likelihood of an individual participant joining a team by demographic characteristics with gender, age category, employment type, BMI, and health status as covariates. Logistic regression was also used for server load analysis. Finally, we used a zero-inflated Poisson (ZIP) regression model to test the association between various predictors (gender, age category, employment type, BMI category, health status, baseline physical activity status, and team participation) and the number of weeks an individual met his or her goal out of eight possible weeks. The choice of a ZIP regression rather than a simple Poisson regression was motivated by the fact that 32% (2,385/7,483) of participants had zero values for meeting goals (i.e. never met goals during any of the eight weeks of the program) (Figure 1). This suggests that there were excess zeros in these outcomes that could not be modeled by a simple Poisson regression. The ZIP model is particularly useful for modeling phenomena where the zeros in the data can be expected to arise from two different sources. For those individuals who did not enter any activity data in a given week, it is impossible to know if they did not meet their goal that week because they did not use the activity log or because they did not do any physical activity that week and thus had nothing to enter. A participant who used the activity log but never had any physical activity to report would appropriately have a zero count for meeting goals. Additionally, a person who signed up for the program but then dropped out and never used the activity log would also have a zero count for meeting goals. It is the former that is typically accounted for by the Poisson counts, and the dropouts are the true source of the excess zeros. A ZIP model is technically a mixture of a usual Poisson model and a mass at zero that attempts to parse out the excess zeros. Fit of a simple Poisson model vs. a ZIP model was tested using the Vuong test statistic [28] without clustering by team. The final ZIP regression results, however, were adjusted for clustering by team.

## Results

### Reach: Program Registration

Of the approximately 47,074 eligible individuals invited to participate in the eight-week Active U program, 7,483 (815 faculty, 5,448 staff, and 1,220 graduate students) registered with the Active U website (≈16% of eligible participants, see Table 1). Although women made up approximately 59% of the eligible participant population, 75% (5,626/7,479) of registered participants were women, indicating that women were more likely to sign up for the program than men. Participants were mostly between the ages of 18 and 59, with 4% (328/7,483) of participants over the age of 60. The majority of participants self-reported their health status as *good*, *very good*, or *excellent*, with only 11% (814/7,482) indicating that their health status was *poor *(1%; 98/7,482) or *fair *(10%; 716/7,482). Participants self-reported an average baseline physical activity of 216 minutes per week (SD = 182 minutes) and 42% (3,106/7,483) were classified as sedentary, as they self-reported engaging in less than 150 minutes of physical activity per week. Of those individuals who provided height and weight information for which BMI was calculated, 49% (2,831/5,777) were overweight or obese with a BMI ≥ 25 (Table 2).

**Table 1:** Recruitment, registration, and physical activity tracker use.

	**Total**	**Faculty**	**Staff**	**Graduate Students**
**University Community**				
**N**	47,074	6,110	26,964	14,000
**% Men**	41%	60%	28%	66%
**% Women**	59%	40%	72%	33%

**Registered for program**				
**N**	7,483	815	5,448	1,220
**% of eligible**	16%	42%	20%	9%
**% Men**	25%	42%	20%	33%
**% Women**	75%	58%	80%	66%

**Entered data at least once**				
**N**	5,885	615	4,478	792
**% of registered**	79%	75%	82%	65%

All faculty, staff, and graduate student members of the university community were eligible to participate in Active U.

**Table 2:** Demographics of Active U participants

	**N**	**%**	**Number of participants that joined a team**	**% of total that joined a team**
**Gender**	**N = 7,479**			
Female	5,626	75%	4,076	72%
Male	1,853	25%	1,270	69%

**Age**	**N = 7,483**			
18 - 29	1,983	27%	1,412	71%
30 - 39	1,781	24%	1,255	70%
40 - 49	1,844	25%	1,341	73%
50 - 59	1,547	21%	1,104	71%
60 - 69	314	4%	227	72%
70+	14	<1%	9	64%

**Employment Type**	**N = 7,483**			
Faculty	815	11%	546	67%
Staff	5,448	73%	3,971	73%
Graduate Students	1,220	16%	831	68%

**Health Status**	**N = 7,482**			
Poor	98	1%	58	59%
Fair	716	10%	470	66%
Good	2,785	37%	1,938	70%
Very Good	2,708	36%	1,982	73%
Excellent	1,175	16%	900	77%

**BMI**	**N = 5,777**			
< 20	381	7%	273	72%
20 - 24.9	2,565	44%	1,867	73%
25 - 29.9	1,712	30%	1,206	70%
30 - 34.9	709	12%	479	68%
≥ 35	410	7%	260	63%

### Effectiveness: Meeting Weekly Goals

The percentage of participants meeting their weekly goals ranged from 43% (3,226/7,483) in week three to 35% (2,612/7,483) in the final week. In total, 11% (815/7,483) of participants met their goals for all eight weeks of the program (Figure 1). Goal attainment peaked in the third week of the program, and the percentage of participants meeting their physical activity goals decreased in subsequent weeks. The third week of the program was the university's spring break. When using a logistic regression adjusting for clustering by team and individual, participants were significantly more likely to meet their goals in week three as compared to week eight (OR = 5.34, *P *< .001).

Through use of the Vuong test statistic, zero-inflated Poisson (ZIP) regression was determined to be more appropriate than simple Poisson regression for analyzing frequency of goal attainment (z = 30.23, *P *< .001). When using the ZIP regression adjusting for gender, age category, employment type, health status, BMI category, baseline physical activity status, and team participation as covariates, several demographic variables were found to be significant predictors of meeting goals. Both graduate students and faculty were less likely to meet their goals than staff members after accounting for excess zeros. Furthermore, older participants met their goals more frequently than the younger participants, while participants with a higher BMI met their goals less often than participants in the normal BMI range. Neither gender nor baseline physical activity status was found to be a significant predictor of meeting goals. Finally, no significant differences were found in meeting goals between those self reporting good health compared to individuals reporting better or worse health states (Table 3).

**Table 3:** Zero-Inflated Poisson analysis predicting participant goal meeting and excess zeros predicted by logistic regression

	**Met Goals**			
	**Zero-Inflated Poisson Regression Predicting Count**		**Predicting Excess Zeros**	
	IRR	*P*	OR	*P*
**Gender**				
Female	Ref		Ref	
Male	0.98	0.25	1.47	< .001

**Age**				
18 - 29	Ref.		Ref.	
30 - 39	1.04	0.24	0.78	0.01
40 - 49	1.12	< .001	0.79	0.02
50 - 59	1.22	< .001	0.67	< .001
60 - 69	1.19	< .001	0.64	0.01
70+	1.44	0.01	0.69	0.54

**Employment Type**				
Faculty	0.93	0.04	1.13	0.24
Staff	Ref.		Ref.	
Graduate Students	0.90	0.04	1.83	< .001

**Health Status**				
Poor	0.90	0.29	1.23	0.46
Fair	1.02	0.60	1.39	0.002
Good	Ref.		Ref.	
Very Good	1.00	0.84	0.90	0.14
Excellent	0.97	0.29	0.97	0.78

**BMI**				
< 20	1.04	0.30	n.s.	
20 - 24.9	Ref.		Ref.	
25 - 29.9	0.95	0.02	n.s.	
30 - 34.9	0.97	0.25	n.s.	
≥ 35	0.90	0.01	n.s.	

**Baseline Physical Activity**				
Sedentary	Ref.		Ref.	
Active	1.00	0.98	1.15	0.07

**Team Participation**				
Individual	Ref.		Ref.	
Team with 2-4 People	1.01	0.92	0.54	0.005
Team with ≥ 5 People	1.28	< .001	0.18	< .001

n.s. = Not Significant

In total, 27% (1,988/7,483) of Active U participants changed their assigned physical activity goal sometime during the course of the intervention with 18% (1,382/7,483) increasing their goal at least once and 14% (1,063/7,483) decreasing their goal at least once. The average weekly physical activity goal across all participants was 216 ± 168 minutes during the first week, and that mean goal did not change substantially over the following seven weeks.

### Adoption: Entering Data into the Activity Tracker

Of the 7,483 people registered, 5,885 people (79%) entered valid data into the physical activity log at least once after their initial registration (Table 1). Participants entered almost 200,000 episodes of physical activity into the activity log. Of the reported episodes, 0.1% (222/199,089), were excluded because they were not valid (<10 minutes or >600 minutes). The percentage of participants entering data each week ranged from 63% (4,742/7,483) in week one to 46% (3,447/7,483) in week eight. In total, 31% (2,304/7,483) of participants entered data for all eight weeks of the program (Figure 1). Participant data entry was highest in week one and subsequent dropout increased over the course of the intervention. When using a logistic regression adjusting for clustering by team and individual, participants were significantly more likely to enter data in week one compared to week eight (OR = 3.82, *P *< .001).

When using the same ZIP regression that predicted number of weeks of meeting physical activity goals adjusted for gender, age category, employment type, health status, BMI, baseline physical activity status, and team participation as covariates, several demographic variables were also found to be significant predictors of excess zeros due to non-participation and/or dropout in the ZIP model. These excess zeros represent participants who failed to adopt the intervention after registering for the program. Specifically, men were more likely than women to have excess zeros, suggesting that they were more likely to drop out. Also, older participants were less likely to produce the excess zeros than the youngest participants, indicating they were less likely to dropout. While graduate students were more likely than staff to have excess zeros, no significant differences between faculty and staff members were detected. Finally, participants self-reporting fair health accounted for more excess zeros than those in good health (Table 3).

### Team Participation

Participants created 460 total teams, ranging in size from 2 to 137 people. Only teams of five or more people competed in the team competition. Approximately 69% (5,182/7,483) of participants were on a competitive team. Table 4 shows team sizes and distribution. In the logistic regression model using gender, age category, employment type, BMI, and health status as covariates, there were no significant associations between age or gender and team participation. When compared to staff members, faculty members were less likely to join teams (OR = .72, *P *= .008). There were no significant differences between graduate students and staff in regards to the likelihood of joining a team. When compared to individuals self-reporting good health status, participants self-reporting very good and excellent health statuses were more likely to join teams (OR = 1.24, *P *= .003 and OR = 1.52, *P *< .001, respectively). BMI was also associated with propensity to join a team as there was a 1% decrease in likelihood to join a team for each one point increase in BMI (OR = .99, *P *= .02). Please refer to Table 5 for logistic regression results clustered by teams for team participation by demographics.

**Table 4:** Distribution of team size

**Team Size**	**Number of teams**	**Number of participants N = 7,483**	**% of participants**
Individual		2,147	29%
2-4	54	154	2%
5-9	193	1,309	17%
10-14	120	1,385	19%
15-19	43	713	10%
20 or larger	50	1,775	24%

**Table 5:** Logistic regression of team participation by demographic characteristics

	**Logistic Regression Predicting Likelihood of Joining a Team**	
	OR	*P*
**Gender**		
Female	Ref	
Male	0.90	0.18

**Age**		
18 - 29	Ref.	
30 - 39	0.92	0.47
40 - 49	0.98	0.89
50 - 59	0.91	0.45
60 - 69	1.03	0.88
70+	0.56	0.41

**Employment Type**		
**Faculty**	**0.72**	**.008**
Staff	Ref.	
Graduate Students	0.75	.17

**Health Status**		
Poor	0.88	0.62
Fair	0.94	0.53
Good	Ref.	
**Very Good**	**1.24**	**.003**
**Excellent**	**1.52**	** < .001**

**BMI**	**0.99**	**0.02**

**Bold **= significant at the .05 level

**Figure 2:**
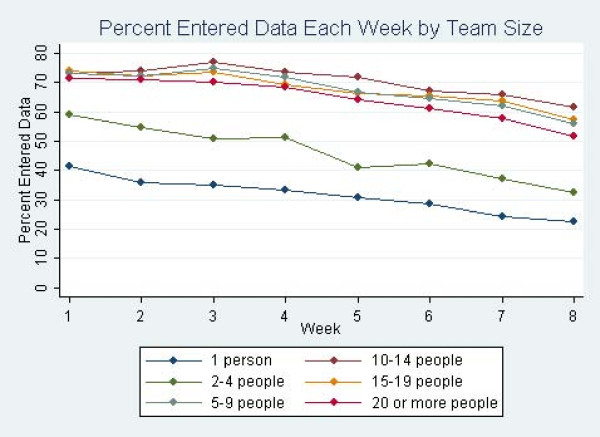
**Percentage of participants entering data per week by team size**.

**Figure 3:**
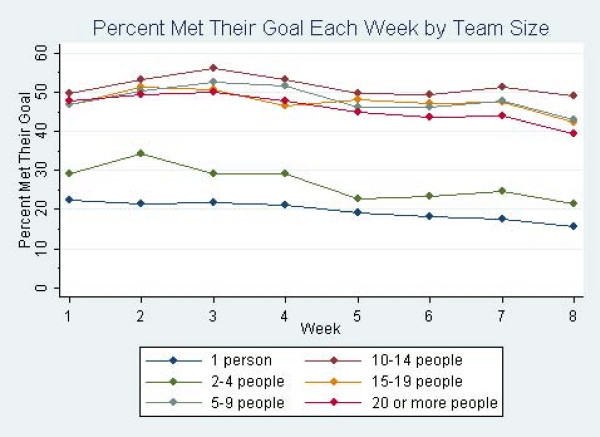
**Percentage of participants meeting goals per week by team size**.

In the ZIP regression analysis, participants who were on any competitive team were less likely to produce excess zeros than people who were not on teams, indicating they were less likely to drop out. Also, the average rate of meeting goals was 28% higher (IRR = 1.28, *P *< .001) among participants who joined a team compared to those who chose not to join a team (Table 3). Figure 2 and Figure 3 show the percent of people who entered data into the physical activity tracker and who met their goals by week and team size. Additional ZIP analyses indicated that there was no optimal team size for meeting goals, as there were no significant differences in rates of meeting goals between competitive teams of different sizes.

### Implementation: The Effect of Server Load on Active U

Finally, it appears that server load issues may have influenced individual participation in the Active U program. Active U server log analysis indicated that median page load times were two to three times slower on Tuesdays than on other days of the week. This was likely due to the sending of weekly emails to all participants on Tuesdays. Comparing all Active U physical activity log sessions that occurred on Tuesdays to ones that occurred on other days of the week, individuals who logged their physical activity on Tuesdays were more likely to drop out of the Active U program than users who logged physical activity on other days of the week (OR = 1.81, *P *< .001).

### Maintenance: The Cost of Active U 2009

The Active U program has been successfully delivered at the University of Michigan for four years. Data from the recently completed 2009 iteration of the program shows that it continues to be a popular program. A total of 9,780 individuals registered with Active U. Program costs have decreased over time as the program builds on previous development and marketing work. The total budget for the 2009 implementation was estimated at $67,580 or a per participant cost of $6.91 per person (see Table 6). In 2007 everyone who registered received an Active U t-shirt and the cost was approximately $45,000. To cut costs and encourage physical activity tracker use, only participants who entered tracker data for a predetermined minimum number of weeks during the program and who also completed the final survey earned t-shirts in 2009. This budget does not reflect the cost of using existing University resources such as email or campus mail, and donated raffle-type prizes from businesses.

**Table 6:** Active U 2009 budget

Web page modifications, maintenance, and server hosting	$26,640
Marketing costs - including design, development, and printing	$10,000
T-shirt incentives for participants	$17,500
Staff costs for customer service and program management	$13,440
Total	$67,580

## Discussion

In terms of reach, the Active U Internet-mediated physical activity program recruited a large number of individuals in a short period of time using a targeted, large-scale advertising campaign. Because enrollment was limited to a well-defined population, it was possible to calculate the percentage of eligible participants who enrolled and succeeded in the program. Of the 47,074 eligible participants, approximately 16% registered with the site.

Reach and adoption rates in this program provide a benchmark for what can be expected in a large Internet-mediated community intervention. Approximately 16% of eligible participants registered and 80% of those used the activity log at least once. This is likely to be at the high end of reach and adoption that we can expect for such programs because of the extensive publicity campaign, the incentives including a t-shirt and gym access for every participant, and the high level of Internet access in the university community targeted by this intervention. Participation in the intervention peaked in the beginning of the intervention and subsequent attrition continued until the end of the eight-week program. Similar findings of attrition and decreased utilization over time for Internet-mediated interventions have been previously reported in the literature [22,29-32]. Future research should focus on determining strategies that will increase participation and adherence throughout the duration of Internet-mediated interventions.

Results suggest that participating in Active U as a member of a team that was eligible for the team competition (i.e., five or more members) increased the effectiveness of the program, even after adjusting for potential confounding by gender, age category, employment type, BMI, and health status. One possible explanation is that team competition created a sense of accountability to others. Additionally, teams may have provided social support, such as encouragement or scheduled group walks. In Active U, participants selected both whether they would join a team and which team they would join. Thus, teams formed around pre-existing affiliations such as departments, buildings, and laboratories. Because both accountability and support are likely to be greater when teams form around pre-existing affiliations, it is possible that teams that formed in other ways, such as random assignment, might not be as effective. If no pre-existing affiliations are available, it may be possible to include program features that would enhance team identity and bonding among team members in order to encourage accountability and social support [33].

Within Active U, although the majority of participants chose to join teams, those individuals who self-reported higher health status, and also had lower BMIs at baseline, joined teams more frequently. Individuals who are heavier or who have poorer health may have chosen not to join a team for a number of reasons. Despite the fact that data on individual participants was not shared among team members in Active U, some participants may have been embarrassed, concerned about confidentiality, or they may have been worried that other team members would not want them on the team because of poor performance. Given the finding that competitive team participation is related to better rates of adherence with Active U, it may be important to encourage team participation among those participants in the highest risk groups.

The Active U recruitment and intervention materials were generic untailored materials and every participant received identical materials. Adding individualized tailoring to both recruitment and intervention materials may improve recruitment of high-risk participants to the intervention and to teams specifically [34]. For example, recruitment emails that specifically address an individual's age or health status might reassure higher risk participants that the intervention is safe and was designed for people like them [21]. Similarly, allowing participants to sign up for common disease-specific newsletters or tips on staying active might increase adoption among higher risk users. Providing more active coaching to allow higher risk users to tailor their physical activity goals may increase goal commitment and encourage participation.

There are a number of technical enhancements that could be made to the Active U website to increase reach and adoption. For example, alternative data entry options such as allowing users to text message physical activity data from a cell phone to the Active U server or allowing users to automatically upload pedometer data from enhanced pedometers may increase physical activity tracker use. Allowing users to view and post their physical activity progress on their personal Facebook page might increase reach and adoption, partly through social marketing effects. Posting physical activity logs and goals publicly or to a network of friends may also improve effectiveness by increasing goal commitment and public accountability. A connection between Facebook and Active U would be particularly important if the program were expanded to include undergraduates, who tend to be frequent Facebook users [35].

Even without adding new ways to enter physical activity tracker data, it is clear from our results that improving website response times might improve adoption. Users who entered activity data on Tuesdays, when the site was slowed down by heavy use, were less likely to enter data in subsequent weeks. Servers should either be provisioned to meet the peak load, or efforts should be made to smooth the load by, for example, sending reminder emails to different people on different days. Using some of the alternative data entry options described previously may also help with server load issues.

Like many other Internet-mediated behavior change programs, women were more likely to participate than men [22,36,29]. While Internet-mediated interventions have been shown to successfully recruit participants, the fact that the majority of participants tend to be women indicates that these programs are failing to effectively reach men who could benefit from these types of interventions. Because of the extensive publicity campaign, it is unlikely that recruitment failed to reach men. Instead, men may have found this type of Internet-mediated physical activity logging program less appealing. Alternatively, men may be less likely to feel that they need help staying physically active.

### Limitations

Active U was not a randomized controlled trial. All participants had access to the Active U intervention, and participants chose whether to participate on a team. Thus, many of the findings reported here may be confounded by individual participant baseline characteristics. While we controlled for some confounding in the multivariate analyses, residual confounding cannot be ruled out. For example, motivation and fitness levels may have been potential confounders that were not measured.

Additionally, we measured physical activity using participant self-reported data in the Active U physical activity log. Self-reported physical activity data correlates poorly with objective measures of physical activity, particularly among those who are more sedentary. If participants did not use the activity log, there was no way to assess their physical activity. In particular, physical activity goal attainment was only captured if participants tracked their weekly physical activity within the Active U system. Individuals who initially registered, but failed to subsequently track their physical activity, might have still achieved their physical activity goals.

Because physical activity data on those individuals who chose not to participate in Active U was not available, it is not known if the program reached those who needed the program the most. The high mean level of baseline physical activity reported in the sample suggests that more sedentary people may have been less likely to register for the program than those who were more active. Finally, the Active U intervention lasted only eight weeks. Even if participants did increase their physical activity during the eight weeks of the program, it is unlikely that this short-term increase in physical activity would translate into long-term health benefits unless the increases were sustained or reinforced in subsequent programs.

## Conclusion

An eight-week, Internet-mediated physical activity intervention that targeted faculty, staff, and graduate students at a large Midwestern university successfully recruited a large number of eligible individuals. Once participants joined the program, about one-third entered physical activity data for all eight weeks of the intervention. Participants who chose to join a competitive team were more likely to meet their goals than participants who chose not to join a team. Internet-mediated physical activity interventions that focus on physical activity logging and goal setting while incorporating team competition may help a significant percentage of the target population maintain their physical activity during the winter months.

## Competing interests

The authors declare that they have no competing interests.

## Authors' contributions

LB, TP, RH, AS, PR, DG, LPD, and CR conceived of the study, participated in its design and coordination, and helped draft the manuscript. LB, RH, AS, and CR performed the statistical analysis. All authors read and approved the final manuscript.

## Pre-publication history

The pre-publication history for this paper can be accessed here:


